# Differences in Human Cortical Gene Expression Match the Temporal Properties of Large-Scale Functional Networks

**DOI:** 10.1371/journal.pone.0115913

**Published:** 2014-12-29

**Authors:** Claudia Cioli, Hervé Abdi, Derek Beaton, Yves Burnod, Salma Mesmoudi

**Affiliations:** 1 Laboratoire d’Imagerie Biomédicale. UMR 7371/UMR S 1146, Sorbonne Universités, UPMC Université Paris 06, Paris, France; 2 ISC-PIF (Institut des Systèmes Complexes de Paris-Île-de-France), Paris, France; 3 School of Behavioral and Brain Sciences, The University of Texas at Dallas, Dallas, United States of America; 4 Sorbonne Universités, Paris-1 Université, Equipement d’Excellence MATRICE, Paris, France; Indiana University School of Medicine, United States of America

## Abstract

We explore the relationships between the cortex functional organization and genetic expression (as provided by the Allen Human Brain Atlas). Previous work suggests that functional cortical networks (resting state and task based) are organized as two large networks (differentiated by their preferred information processing mode) shaped like *two rings*. The first ring–Visual-Sensorimotor-Auditory (VSA)–comprises visual, auditory, somatosensory, and motor cortices that process real time world interactions. The second ring–Parieto-Temporo-Frontal (PTF)–comprises parietal, temporal, and frontal regions with networks dedicated to cognitive functions, emotions, biological needs, and internally driven rhythms. We found–with correspondence analysis–that the patterns of expression of the 938 genes most differentially expressed across the cortex organized the cortex into two sets of regions that match the two rings. We confirmed this result using discriminant correspondence analysis by showing that the genetic profiles of cortical regions can reliably predict to what ring these regions belong. We found that several of the proteins–coded by genes that most differentiate the rings–were involved in neuronal information processing such as ionic channels and neurotransmitter release. The systematic study of families of genes revealed specific proteins within families preferentially expressed in each ring. The results showed strong congruence between the preferential expression of subsets of genes, temporal properties of the proteins they code, and the preferred processing modes of the rings. Ionic channels and release-related proteins more expressed in the VSA ring favor temporal precision of fast evoked neural transmission (Sodium channels SCNA1, SCNB1 potassium channel KCNA1, calcium channel CACNA2D2, Synaptotagmin SYT2, Complexin CPLX1, Synaptobrevin VAMP1). Conversely, genes expressed in the PTF ring favor slower, sustained, or rhythmic activation (Sodium channels SCNA3, SCNB3, SCN9A potassium channels KCNF1, KCNG1) and facilitate spontaneous transmitter release (calcium channel CACNA1H, Synaptotagmins SYT5, Complexin CPLX3, and synaptobrevin VAMP2).

## Introduction

In order to understand how the human cerebral cortex processes information we need to connect the anatomo-functional organization of the cortex (as described, e.g., in [1]–[3]) with the topographic organization of gene expressions [4]–[6]. To do so, several recent studies have analyzed–in species such as monkey, mouse, and human [7]–[9]–the pattern of genetic expression of cortical regions (e.g., visual or prefrontal cortices). These studies revealed that cortical regions differ in the genes that they express. This pattern can be seen, for example, in mouse brain atlases of gene expression [10]–[14]. In humans–because the strong constraints of postmortem genome-wide analysis make the data hard to obtain–only very few studies [7], [15] have explored the systematic anatomic organization of genetic expression across the whole brain.

**Figure 1:**
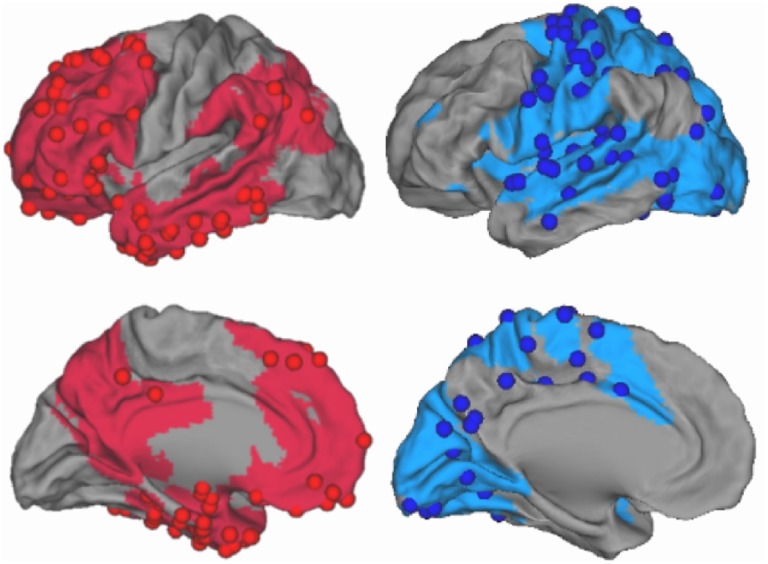
The two intertwined rings corresponding to different type of temporal processing. The VSA ring (in blue) corresponds to high fidelity evoked processing and the PTF ring (in red) corresponds to more spontaneous processing more independent of input action potentials timing (from [3]). Cortical regions sampled by the Allen Institute for Brain Sciences ([7]) are represented by spheres colored like their respective rings. Points in sulci are not visible.

By contrast with this small number of genetic systematic analyses, there are several systematic analysis of anatomo-functional cortical networks and functional connectivity as revealed by brain imaging. Together these studies show (as supported by several meta-analyses) that the correlations between task based networks (TBN), resting state networks (RSN), and anatomical networks (AN) are particularly robust [2], [3], [16], [17]. So, both the cortical expression of genes and the functional networks have been explored, but no attempt has been made, so far, to relate the information processing performed by cortical functional networks and the specific cellular properties of proteins coded by genes expressed in these networks. In this paper, we focus on the functional differences observed at large scales between networks which directly process the interaction with the external world and networks which are more internally driven. This large scale functional dichotomy–taken into account also in [18]–[21]–has been systematically analyzed and modeled in [3] where we showed that task TBNs, RSNs, and ANs could be integrated into a common model called the “dual intertwined ring architecture” (Fig. 1). Although this architecture had not been previously discussed as such, most published results dealing with the organization of RSNs present patterns that are compatible with it [22]–[24].

According to this “dual ring” model, the human cerebral cortex comprises two large structures shaped like rings. The first ring–called the Visual-Sensorimotor-Auditory (VSA) ring–is continuous and forms a circle around parietal areas BA 39 and 40, whereas the second ring–called the Parieto-Temporo-Frontal (PTF) ring–is not fully continuous over the cortical mantle but is closed by long-range association fiber tracts (longitudinal parieto-frontal, arcuate, uncinate, and cingulum) that complete the intertwining. The two rings share a set of common regions mostly localized along the precentral, intraparietal, and superior temporal sulci. The correlation of RSN and TBN as described in [2], [17] shows that VSA regions integrate various sources of auditory, visual, and somatomotor information together. VSA regions also perform bimodal interactions: between visual and motor (e.g., grasping, reaching, imitation) between auditory and somatomotor information (e.g., recognizing and producing phonemes) and between auditory and visual information (important for communication). All these functions imply strong real time constraints.

A similar comparison with RSNs and TBNs shows that the PTF ring implements at least four main groups of functions [2], [3], [17]: (1) biological regulation, olfaction, taste, and emotion which operate at different time scales, [2] (2) working memory and attention: planning and working memory (in the lateral frontal network of the PTF ring) which are based on the integration of information across time by sustained neuronal activations and are able to integrate in the same sequence, several sensory and motor events separated by long and variable delays (reviewed in [25], [26]), (3) self-referential functions and social cognition which require information to be integrated at different temporal scales from the past (memory), the present and the future [27]–[31] and (4) language which necessitates integration of information at different time scales (words, sentences, story)[32].

By contrast with the real-time information processing of the VSA ring, we propose that the functions implemented by PTF are produced by “multi-temporal integration” processes. The dual process that we postulate across the two rings (i.e., real-time and multi-temporal integration) is close to the dual process proposed in [33], [34] with both perception-action cycles and working memory (WM) processes. Evidence for this regional temporal specialization has also been found in experiments using single cell recording in non-human primates [35] and in empirically based mathematical models of the human cortex [36]. Furthermore, evidence from brain imaging and brain lesions indicates that spontaneous activity characterizes regions belonging to PTF. For example, in [28] the authors report–from brain imaging experiments–that regions corresponding to a large portion of PTF (specifically the “default mode network”) are spontaneously active even when no task is performed. Similarly patients affected by lesions within the dorsolateral prefrontal cortex suffer loss of spontaneous motor activity and spontaneous language production, a pattern often described as the “frontal syndrome” (see review in [25], [26], [37]).

The comparison of the sensorimotor and cognitive functions implemented by the two rings shows–in agreement with the literature cited above–that the two sets of functional networks differ essentially in the way they process information: The functional networks forming the VSA ring process real time information whereas the functional networks forming the PTF ring process multi-scale temporal information with a wider time range and also generate spontaneous activity. We expect that the difference between the processing modes of the rings will be reflected in the dynamics implemented in their populations of neurons. For example, neuron activation can be evoked by incoming action potentials with high temporal precision or conversely spontaneous and more independent on timing of input action potential. High temporal precision and strongly input driven dynamics should be favored in the VSA ring while more spontaneous, sustained, and rhythmic activity should be favored in the PTF ring. These different types of information processing are likely to be implemented by families of proteins regulating ionic currents [i.e., potassium channels (KCNs), sodium channels (SCNs), and calcium channels (CACNs)] and neurotransmitter release (such as, e.g., synaptotagmins or synaptobrevins). We thus expect–in accordance with their information processing specialization revealed by brain imaging studies–to find differences in the expression of genes coding for ionic channels and transmitter release in the VSA and PTF rings.

In the present study, we explore the differential expression of genes into the two rings and the differential properties of related proteins critical for information processing. To estimate cortical genetic expression we used the Allen Human Brain Atlas (ABA) [38] of the human transcriptome–produced by the Allen Brain Institute–which provides microarray expression profiles of almost every gene of the human genome at hundreds of locations in the brain for two complete postmortem brains. Fig. 1 shows the localization of ABA regions with respect to the two rings topography. In this paper:

(1) we used correspondence analysis (CA) to describe the pattern of expression of the 938 genes with the largest variance of expression (as in [7]) and found that the first dimension of this analysis opposes two large sets of regions; (2) we show that these two sets of regions representing the major source of genetic variation match the topography of the two rings; (3) we corroborate the results of (1) and (2) by showing that each cortical region can be reliably associated with one of the two rings based on its profile of genetic expression; (4) we show that genes are organized into two exclusive groups that reflect the functional opposition between the two rings; (5) we show that many proteins coded by genes that most differentiate between cortical regions belong to families of proteins involved in neuronal information processing such as ionic channels and neurotransmitter release; (6) we show that the systematic study of families of genes identified in (5) (e.g., ionic channels) reveals the proteins most expressed in each of the two rings; and (7) we show that the temporal properties and neural dynamics of these proteins closely correspond to the information processing differentiated in the two sets of functional networks.

To obtain these results we used multivariate analysis techniques. First, we used CA [39]–[43] to describe the patterns of genes expression over the cortex. This multivariate descriptive technique classifies the regions based exclusively on their expression profile and does not take into account their topographical position (e.g., if they belong to the VSA or PTF areas or not). Second, we used discriminant correspondence analysis (DiCA) [44]–a discriminant analysis method derived from CA–to quantitatively evaluate whether the differences in gene expression across cortical regions are informative enough to predict the ring membership of these regions. We also used DiCA analysis to identify the genes that most contribute to the differentiation between the rings.

## Materials and Methods

### Data Base

The data used in this paper were obtained from the Allen Brain Atlas project [38]. We used for this study two postmortem brains (H0351.2001 and H0351.2002) that were sampled for (respectively) 946 and 896 distinct brain locations representing all structures within the brain in approximate proportion to the volumetric representation of each cortical, subcortical, cerebellar, and brain stem structures. From these two sets of regions, we selected the subsets of: (1) all 394 cortical regions in the first brain, and (2) all 337 cortical regions in the second brain. The genetic expression of the cortical regions was described by the subset of 938 genes selected in [7] for their ability to generate the largest differences among 56 groups of regions of the cortex. In this paper, we present only the analysis performed on Brain H0351.2001 because the analysis performed on Brain H0351.2002 provided highly similar results (the results for Brain H0351.2002, however, can be found in the Supporting Material).

### Labeling of cortical regions by VSA or PTF rings

The 394 (for Brain H0351.2001) and 337 (for Brain H0351.2002) cortical regions used in this study were identified from their MNI (Montréal Neurological Institute; [45]) coordinates which were then used to assign each region to one of two groups of regions: ring VSA or ring PTF according to the labeling scheme described in [3].

### Statistical Analysis I: Correspondence Analysis (CA)

In order to describe cortical genetic organization we used correspondence analysis (CA) [39]–[42]. CA–a variant of principal component analysis (PCA)–is used when the data are non-negative numbers (as is the case here, see [43], [46]) and when the goal is to compare observations according to the relative distributions–as opposed to the absolute values–of a set of variables. Here, observations are brain regions and variables are genes, as measured by their expression values. To do so, for each region, the genetic expression values are scaled such that their sum is equal to one (this re-scaled vector of genetic values for a given observation is called a *profile*) and a generalized PCA (i.e., a generalized singular value decomposition, see, e.g., [39], [42], [47]) is then applied to the profile data matrix. This generalized PCA creates–from both observations and genes–a new set of orthogonal variables, called factor scores (a.k.a. components or dimensions), that best represents the variability in the original data. Observations and variables are assigned scores–called factor scores–on the factors. These factor scores can be used to create―for both cortical regions and genes―maps in which the proximity between items reflects the similarity of their profiles. To better identify the genes consistently associated with a given dimension, we also computed bootstrap ratios (see [48], [49]). To do so, we generated 1,000 new bootstrapped samples by sampling with replacement from the set of cortical regions. The factor scores for the variables of each bootstrap sample were then computed as supplementary (a.k.a., “out of sample”) variables (this procedure is sometimes called “partial bootstrap,” see, e.g., [39]). For each gene, the mean and standard deviation of these factor scores were then computed and a statistic akin to Student’s *t*–called a bootstrap ratio (denoted *BT*, see, [49], for details)–was then computed by dividing the mean bootstrapped factor score of this gene by its standard deviation. By analogy with a *t*-statistic, a bootstrap ratio value of 2.00 will (roughly) correspond to a (un-corrected for multiple comparisons) *p* value of.05. Using a Bonferroni correction [50] for multiple testing for 938 comparisons (i.e., the number of genes) will give a corrected value of 3.90 that we rounded to 4.00. We used the implementation in the [R] programming language (R Development Core Team, 2013) of CA obtained from the packages ExPosition and InPosition (see [47] and http://cran.r-project.org/web/packages/ExPosition/index.html). We also used the R packages Hmisc (http://cran.r-project.org/web/packages/Hmisc/index.html) and beeswarm (http://cran.r-project.org/web/packages/beeswarm).

### Statistical Analysis II: Discriminant Correspondence Analysis (DiCA)

In order to test our hypothesis that the profile of genetic expression of a region depends upon its ring (i.e., VSA or PTF), we used discriminant correspondence analysis (DiCA)–a discriminant analysis version of CA [44]–to predict the regions’ ring from their gene expression profiles. We used the implementation of DiCA in the TExPosition/TInPosition packages (http://cran.r-project.org/web/packages/TInPosition/index.html; see also [47] and R Development Core Team, 2013). DiCA creates a matrix that represents each group (i.e., PTF and VSA) by the sum of its observations (for all the gene expressions). In our case, this matrix is a 2×938 (2×161 for the second pool of genes analyzed) data table that contains the sum of each gene expression level (columns) for each ring (rows). This data table is then analyzed by CA (this step solves the discriminant problem because CA computes factor scores that maximize the difference between rows). The original cortical regions are then projected as supplementary elements (a.k.a. “out of sample”) in the group factor space and the distance of each cortical region to each ring is also computed in this space [39]. Finally each cortical region is assigned to its closest ring. The quality of this assignment can be assessed by 1) a confusion matrix that shows for each ring the number of cortical regions of this ring assigned to this and the other ring and 2) by an *R* ^2^ statistic computed as the ratio of the ring variance to the total variance of the cortical regions. A random effect confusion matrix is also computed using a leave one out cross-validation scheme: For each cortical region, the whole analysis is run without this region which is then projected (as a supplementary element) in the ring factor space and assigned to the closest ring. The quality (i.e., “significance”) of the *R* ^2^ is evaluated by computing a probability distribution under the null hypothesis with a permutation test: For 1,000 iterations, the labels of the cortical regions are shuffled and the *R* ^2^ is recomputed. In addition, a permutation test was also used to evaluate the null hypothesis of an overall effect and to identify significant components (see [47]). Also, the bootstrap resampling scheme used in CA (obtained by resampling the cortical regions with replacement) was also used in DiCA to compute bootstrap ratios for the genes and also to derive confidence intervals for the groups. An equivalent bootstrap resampling scheme was used to compute bootstrap ratios for the regions. To further assess the quality of the assignment model, we also used the model computed on one brain to assign to their rings the brain regions of the other brain.

## Results

For this study, we replicated all results by performing the same analysis on two different postmortem brains for which a similar genome-wide analysis was performed. We found that the conclusions were highly similar (see also [7]), thus for simplicity we present in details only the results for Specimen H0351.2001. Complete results about Specimen H0351.2002 are reported in the Supporting Information.

### Gene expression spontaneously separates the rings

We first performed a descriptive multivariate analysis of the topographic distribution of genetic expression over the cortical surface. This analysis can reveal if there is an underlying organization of these expressions. Specifically, we used CA (see Materials and Methods) to describe the relationships between 394 cortical regions and the expression of 938 genes that were selected by [7] because these genes had the largest variation in expression across the cortical surface (see Materials and Methods section). CA (a multivariate descriptive method) computes–without about using the a-priori information about ring membership–the similarity between cortical regions based on their profiles of genetic expression and represents the configuration of the similarity between these regions along dimensions (also called factors) that maximize the variance between these cortical regions. CA also represents, in the same space, the similarity of the genes derived from their profiles of expression over the regions (see [39]).

**Figure 2:**
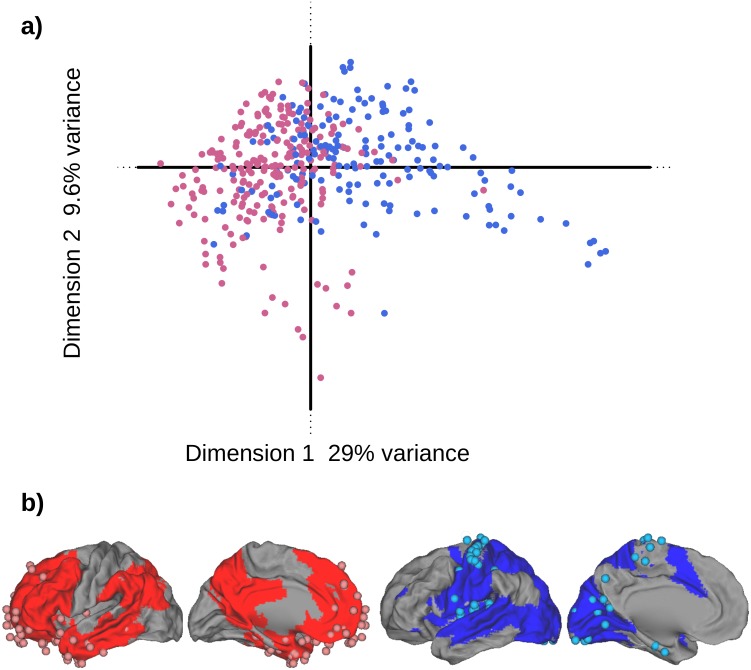
Differential distribution of gene expression: CA analysis. **a)** Dimensions 1 and 2 as extracted by a CA performed on the 394 regions and the 938 genes. The dots represent the factor scores for the regions; each dot is colored in red or blue depending on the represented region localization within (respectively) PTF or VSA. The eigenvalue of Dimension 1 (λ_1_ = 4.59^–03^) represents 29% of the total variance, The eigenvalue of Dimension 2 (λ_2_ = 1.51^–03^) represents 9% of the total variance. **b)** The localization of the cortical regions on the brain with PTF and VSA rings colored in, respectively, dark red and dark blue. Light red dots represent regions with significant negative factor scores (*BT*<–2.00) whereas light blue dots represent regions with significant positive factor scores (*BT*>2.00).

The analysis revealed that a large proportion (i.e., 47%) of the explained variance was concentrated on the first four dimensions that explained respectively: 29%, 9%, 5%, and 4% (*p*<.001, .001, .001, .001, respectively by permutation test) of the total variance. Fig. 2a displays the map obtained by plotting the region factor scores for the first 2 dimensions of the analysis. This graph shows that there are two different sub-sets of cortical regions opposed on the first dimension. In Fig. 2a, each region is represented by a dot colored according to its topographical membership to the VSA ring (in blue) or to the PTF ring (in red) to indicate if the two ring structure was related to the first dimension of CA. Fig. 2a shows that PTF regions are clustered on the left side of Dimension 1 (negative factor scores), whereas most of the VSA regions are clustered along the right side of Dimension 1 (positive scores). When we plot the highly significant regions (using their bootstrap ratios, see Materials and Methods) on the cortical surface (Fig. 2b) we find that the two sub-sets show a clear match between the first dimension and the PTF and VSA rings. This configuration of the regions on Dimension 1 indicates that the patterns of gene expressions spontaneously organize the cortical regions in two groups that closely correspond to the VSA and PTF rings (note that this result is obtained without using the information about the regions anatomical position or ring assignment). Thus, the functional model in two rings appears to be the primary source of cortical genetic variation.

This result is confirmed by a similar analysis performed on Brain H0351.2002. Here again, the first dimension of the CA clearly opposes two ensembles of regions that match the two rings (S1 Fig.). Region factor scores for Dimensions 1 and 2 and region bootstrap ratios for Dimension 1–for Brains H0351.2001 and 2 – are reported as supporting material in S1 and S2 Tables.

### Cortical gene expression predicts the assignment of cortical regions to the rings

In the previous section, we showed that the first dimension of CA identified two distinct subsets of cortical regions. These subsets closely match the PTF and VSA rings. The large proportion of variance explained by Dimension 1 of the CA indicates that the different profiles of genetic expression are associated with different rings, but is this large association strong enough to actually predict the ring membership of cortical regions? For the present analysis, we labeled each cortical region according to its membership to the PTF or VSA rings (following the scheme described in [3]) and used a discriminant analysis version of CA–DiCA–to assign each region to a ring. To assign a region to its ring, DiCA combines the values of gene expression to create new optimal dimensions that best discriminate these two groups of regions and also identify the genes responsible for this separation.

**Figure 3:**
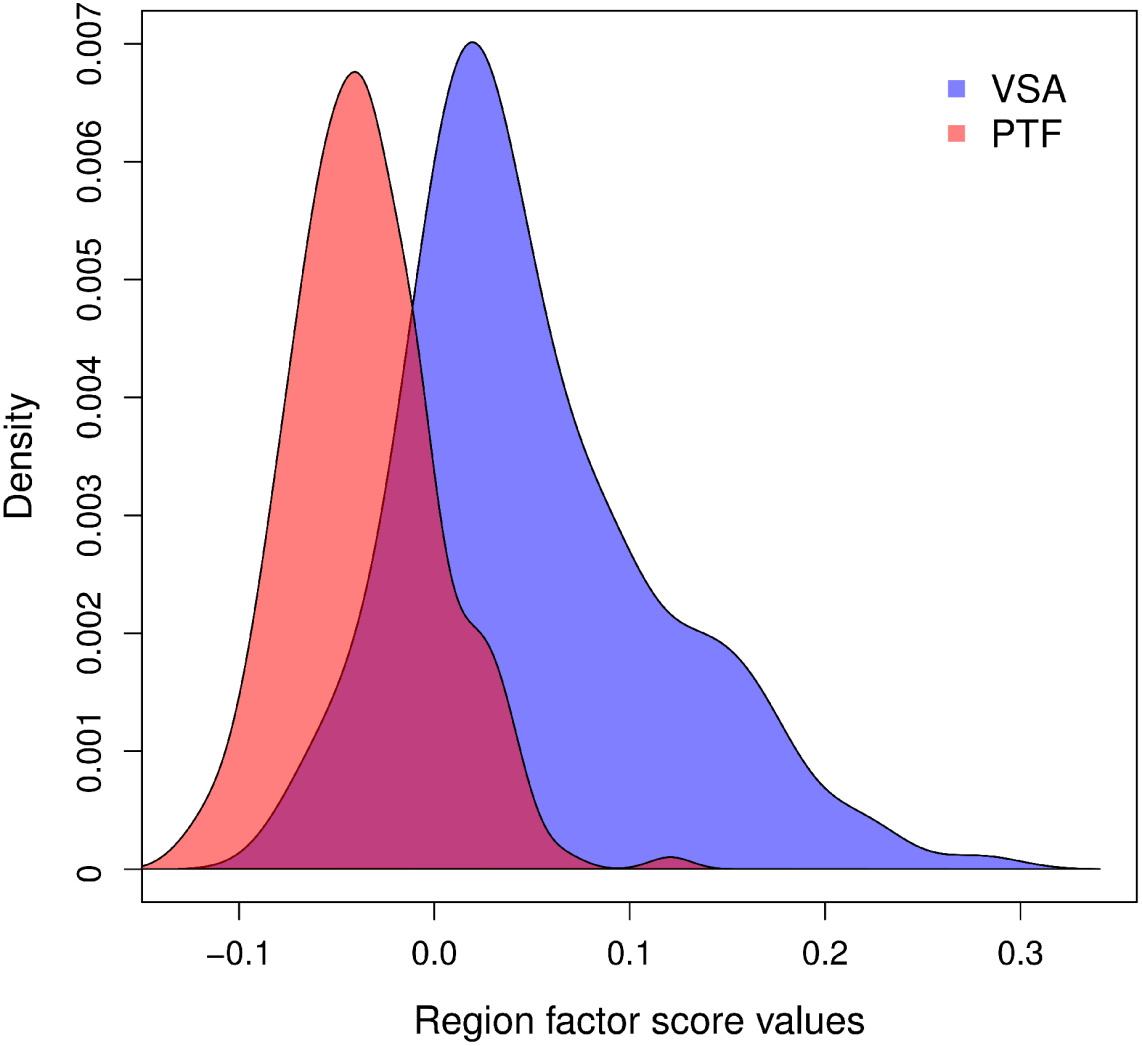
DiCA analysis: regions factor scores histogram. We plot the histogram of the factor score values–obtained for the 394 regions by the DiCA analysis–as a function of the number of regions *a priori* assigned to the VSA (blue) or the PTF (red) ring.

With two groups, DiCA extracts only one dimension. In addition to separating the two a priori groups (PTF and VSA, see Fig. 3 for Brain H0351.2001 and S2 Fig. for Brain H0351.2002), DiCA also provides information on the assignment–predicted from gene expressions–of the cortical regions to these groups. As can be seen in S3 Table, 320 out of the 394 cortical regions were correctly classified (i.e., 81%, *p*<.0001 by Binomial test; for Brain H0351.2002, cf. S4 Table, we found 315 out of 337 regions correctly classified, or 93%, *p*<.001, by Binomial test). To insure that this performance was genuine, we also used a random effect model based on a leave one out procedure (see Materials and Methods section, see, also results in S5 Table for Brain H0351.2001) and found that we could then correctly assign 319 out of 394 regions (i.e., 81%, *p*<.0001 by Binomial test, for Brain H0351.2002, cf. S6 Table, we found 313 out of 337 regions correctly classified or 93%, *p*<.001, by Binomial test). All these results clearly confirm that the genetic profile of a cortical region can determine its localization within one of the two functional rings.

### Correlation between gene expressions

**Figure 4:**
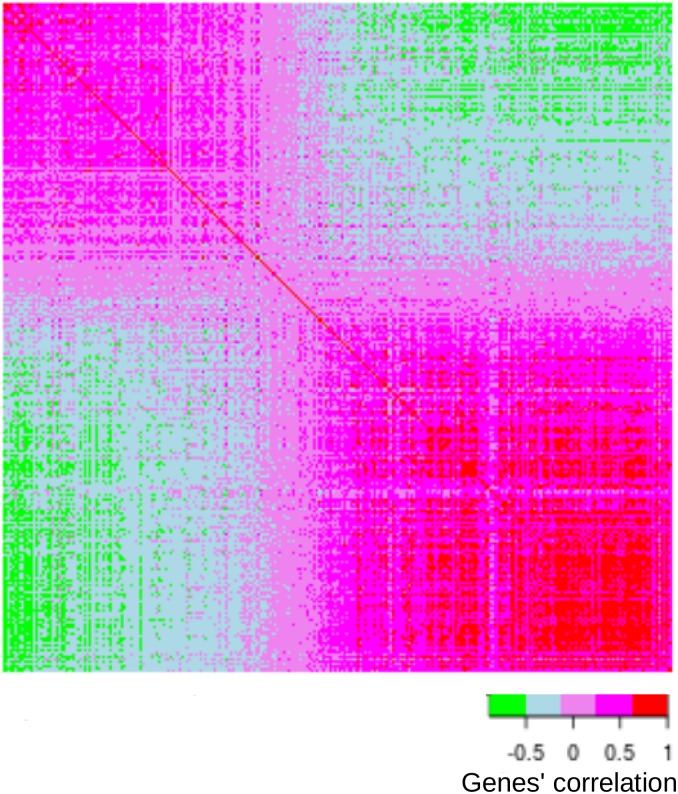
Heatmap representing the correlations between the 938 genes used in DiCA. The correlation coefficients between genes were computed using all 394 regions. The genes are ordered according to their positions on the dimension extracted by DiCA. Red to magenta colors denote strong positive correlations whereas green denotes a strong negative correlation. The genes are clearly organized into two blocks that are related to the gene differential expression for the two rings.

To better understand the organization of the genes between and within the rings (along the first dimension), we computed the correlations (across the regions) between genes. To graphically represent these correlations we ordered the genes according to their position on Dimension 1 of the DiCA (see S7 Table) and colored the correlations according to their values (see Fig. 4). As can be seen in Fig. 4, the correlations (outside the diagonal) ranged from –.89 to +.96 and the genes were clearly organized into two exclusive blocks with genes positively correlated within each block and negatively correlated between blocks. This opposition reflects, at the gene level, the functional opposition between the two rings.

### The proteins coded by genes most differentiated between cortical regions belong to families of proteins involved in neuronal information processing such as ionic channels and neurotransmitter release

**Figure 5:**
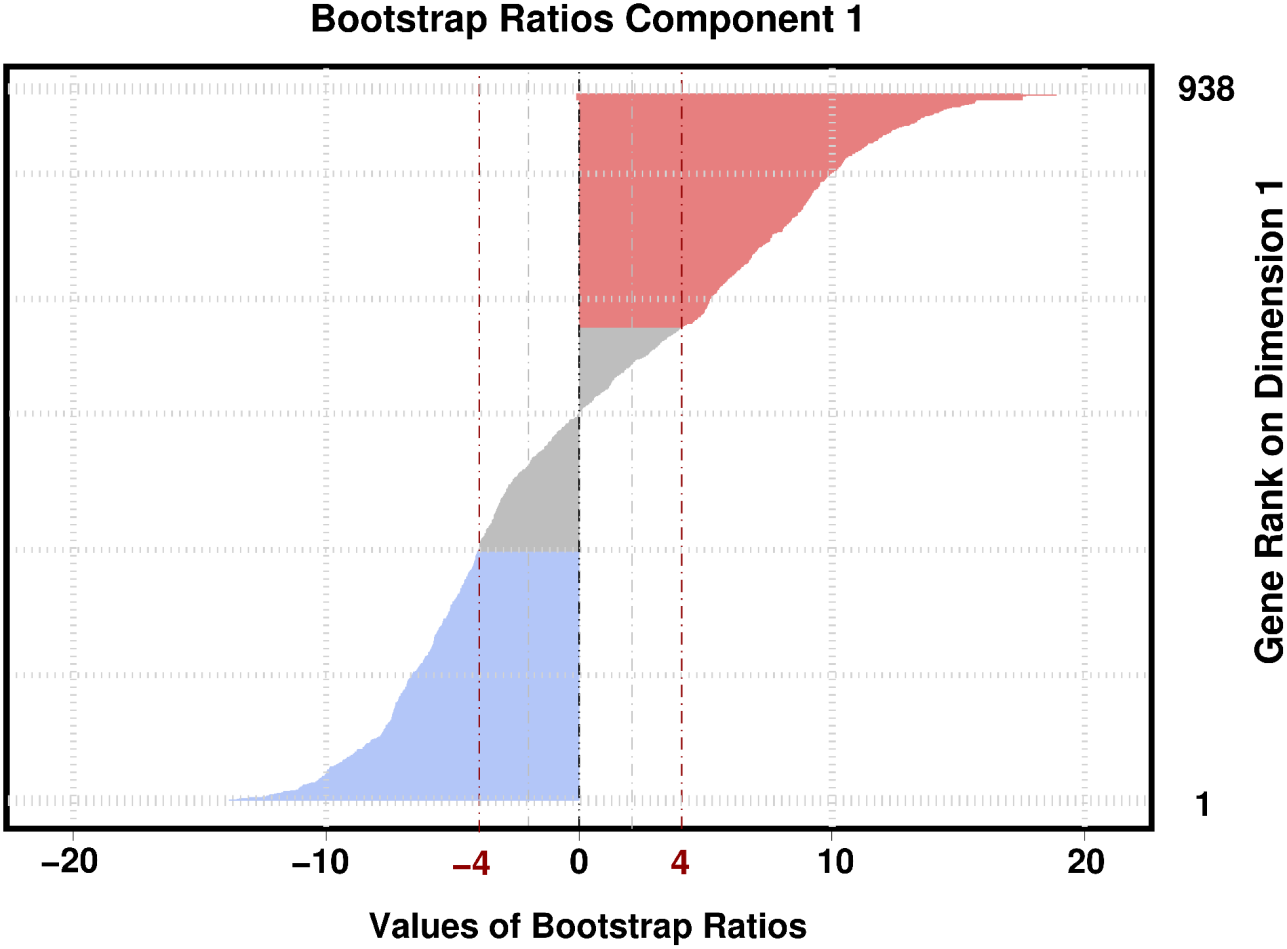
Bootstrap ratios for the genes for the Dimension extracted by DiCA. A very large proportion (i.e., 66.5%) of the genes are significant with |*BT*|>4.00. This indicates that the expression of most genes differ significantly between regions.

In order to identify the genes important to discriminate between PTF and VSA rings, we also computed bootstrap ratios for the genes (see Fig. 5, see also S7 and S8 Tables for the complete list of genes and their bootstrap ratios for the two brains). Out of 938, we found that 650 (i.e., 69.2%) genes had a significant bootstrap ratio (*BT*) for the first dimension (a value of *BT*>4.00 roughly corresponds to a Bonferroni/Šidák corrected value of *p* = .05 for 938 comparisons, see [50]).

Among the genes that mostly differentiate between the two rings, we found genes that code for proteins important for neuronal functions such as ionic channels, neurotransmitter release, receptors, cytoskeleton, transcription factors, and cellular recognition. Specifically, associated with the VSA ring, we found genes coding for sodium channels isoforms such as SCN1A (*BT* = 15.81) and SCN1B (*BT* = 16.54) and genes coding for potassium channels isoforms such as KCNA1 (*BT* = 14.98). By contrast, associated with the PTF ring, we found KCNG1 (*BT* = –14.06) that codes for a potassium channel, and CACNA1H (*BT* = –10.13) a gene that encodes syntaxin-1A/Ca(v)3.2 T-type calcium channel. Along the same lines, we found strongly associated with VSA, genes encoding for proteins implied in neurotransmitter release such as SYT2 (*BT* = 13.58), CPLX1 coding for Complexin 1 (*BT* = 12.42) that influences the fast kinetics of cellular recognition release (i.e., “kiss-and-run”). We found also VAMP1 encoding for Vesicle-Associated Membrane Protein (*BT* = 17.12) strongly associated with the VSA ring and important for maintaining the pool of vesicles for high frequency stimulus driven release.

Among the other families identified by their high bootstrap ratios we also found glutamate receptors (GPRIN1, *BT* = –8.56; GRM1, *BT* = –7.78), hormone receptors (ESRRG, *BT* = 16.31; PMEPA1 *BT* = 11.84), neuropeptides (GRP, *BT* = –9.45; TAC1, *BT* = –7.66), kinases (PLCD4, *BT* = –15.40; PRKDCD, *BT* = –11.44), transcription and growth factors (EGFR, *BT* = 15.29; ASCL2, *BT* = 11.54), genes implicated in cell communication (SSTR1, *BT* = –9.63; CORT, *BT* = –9.29) and associated to cytoskeleton (RPGR, *BT* = 12.42; CORO1A, *BT* = –8.13).

### Determination of genes the most expressed in VSA and PTF for the ionic channels and transmitter release functional classes

Among the most extreme genes (i.e., the genes most important for the opposition between VSA and PTF) revealed by our analysis, we found, in particular, genes coding for proteins responsible for two classes of neuronal functions that are directly implicated in the shaping of neuronal dynamics and with specifically known time properties: ionic channels and neurotransmitter release. Genes coding for proteins responsible for these two classes of neuronal functions appear to be distributed on both the VSA and PTF regions. To better understand the characteristics of this distribution and the specific connection with either VSA or PTF regions, we performed a systematic analysis on these two groups of genes. Table 1 shows the 6 families of genes pertaining to these two classes of neuronal functions for a total of 161 genes. Table 1 also lists the number of genes per family and the number of genes per class.

**Table 1:** The 161 genes coding for proteins forming ionic channels or involved in neurotransmitter release.

Neuronal function class	Family name	Family symbol	*N* genes per family	*N* genes per neuronal function class
Ionic channels	Potassium channels	KCN	94	135
	Sodium channels	SCN	15	
	Calcium channels	CACN	26	
Neurotransmitter release	Synaptotagmins	SYT	16	26
	Complexins	CPLX	6	
	Synaptobrevins	VAMP	4	

In the analysis of the genes the most differentially expressed on the cortex we found genes belonging to ionic channels and neurotransmitter release neuronal function classes. In particular these genes belong to six families of genes (KCN, SCN, CACN, SYT, CPLX, and VAMP). The members of these six families (when we take all the members for each family) sum up to a total of 161 genes. The table gives the number of genes per family and the number of genes per class.

We first verified that this pool of 161 genes was able to discriminate regions belonging to the two rings. To do so we used these 161 genes to perform a DiCA predicting the ring membership of the 394 regions from Brain H0351.2001. Results (S9 Table) indicate that DiCA correctly predicts the ring membership of 324 out of these 394 regions (82%, *p*<.0001 by binomial test). As we did for the set of 938 genes, to insure that this performance was genuine, we also used a random effect model based on a leave one out procedure (see Materials and Methods section, see, also results in S10 Table) and found that we could then correctly assign 321 out of 394 regions (i.e., 81%, *p*<.0001 by binomial test). For Brain H0351.2002 (cf. S11 and S12 Tables) we found, for the fixed and random effects, values of (respectively) 306 and 305 (out of 337) correctly classified regions (91% and 90%).

**Figure 6:**
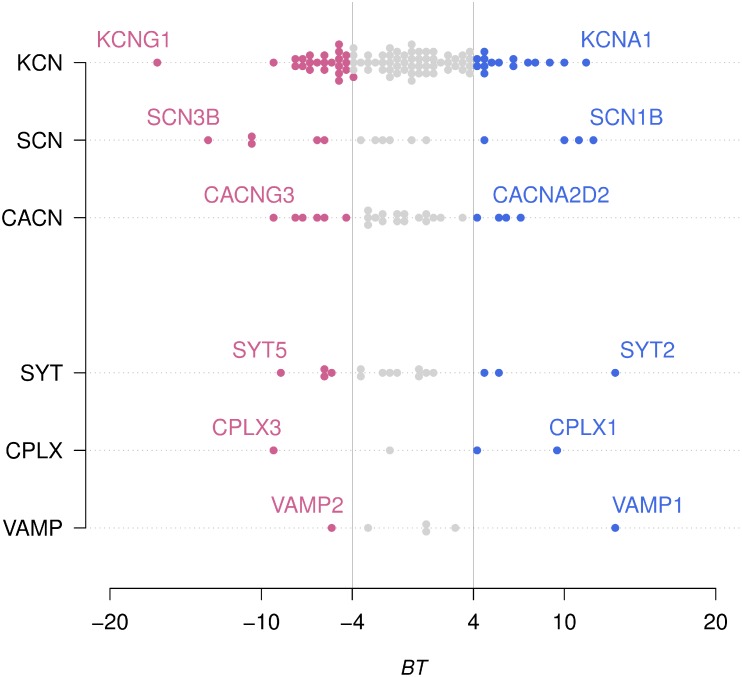
Map of the bootstrap ratios for the 161 genes, analyzed with DiCA, grouped per family and per class of neuronal function. In blue are represented genes with significant positive bootstrap ratios (*BT*>4.00) associated with the VSA ring and in red genes with significant negative bootstrap ratios (*BT*<–4.00) associated with the PTF ring. For each family, extreme genes are identified. These genes are the most preferentially expressed in either VSA or PTF.

To further assess the predictive quality of the DiCA model, we also used the model derived on one brain to predict the ring assignment of the regions from the other brain (i.e., a “one brain left out” scheme). Doing so, we were able to correctly assign 287 regions out of 394 for Brain H0351.2001 (i.e., 73%, *p*<.0001 by Binomial test) and 288 regions out of 337 for Brain H0351.2002 (i.e., 85%, *p*<.0001 by Binomial test). Fig. 6 shows the results for the bootstrap analysis separating, on each row, the results pertaining to a single family. Represented in blue are genes with significant positive bootstrap ratios (*BT*>4.00, indicating a preferential expression in VSA) while in red are genes with significant negative bootstrap ratios (*BT*<–4.00, indicating a preferential expression in PTF). For each family, extreme genes (i.e., best predictors) are labeled.

**Figure 7:**
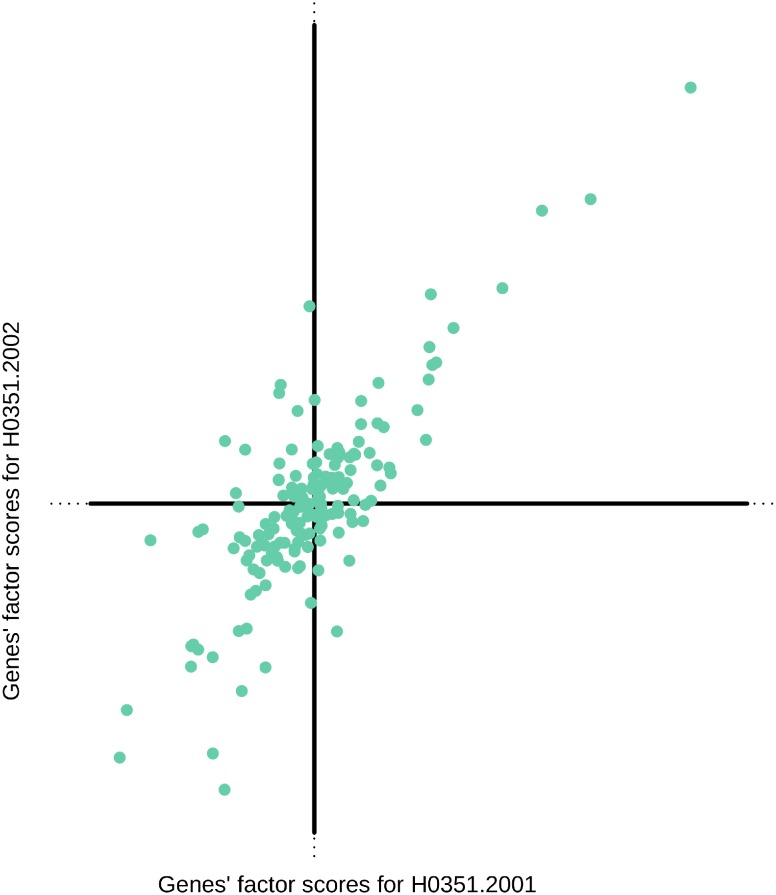
Scatter plot of the gene factor scores of the discriminant dimension extracted by the DiCA analyses performed on the 161 genes measured on Specimens H0352001 (horizontal) and H0352002 of ABA. Each dot represents one of the 161 genes. The coefficient of correlation is equal to .90 (*r* ^2^ = .81, *p*<.001).

Similar results for Brain H0351.2002 are reported in S3 Fig. It is interesting to note that, for each family, extreme genes are essentially the same in both brains (see S13 Table for a complete list of bootstrap ratios). To better quantify the reproducibility of the results of the DiCA in the second brain we plotted against each other the gene factor scores obtained by the DiCA analysis of Brains H0351.2001 and H0351.2002. Fig. 7 illustrates the high correlation between the results of these two brains (*r = *.90, *r* ^2^ = .81, *p<*.001). Moreover we found that, for each family under investigation, the most significant PTF or VSA genes extracted by their bootstrap ratios were the same in almost all cases (compare Fig. 6 with S3 Fig.). When this was not the case, the genes that had an extreme bootstrap ratio for the first brain had the second most extreme bootstrap ratio for the second and were still highly significant.

**Figure 8:**
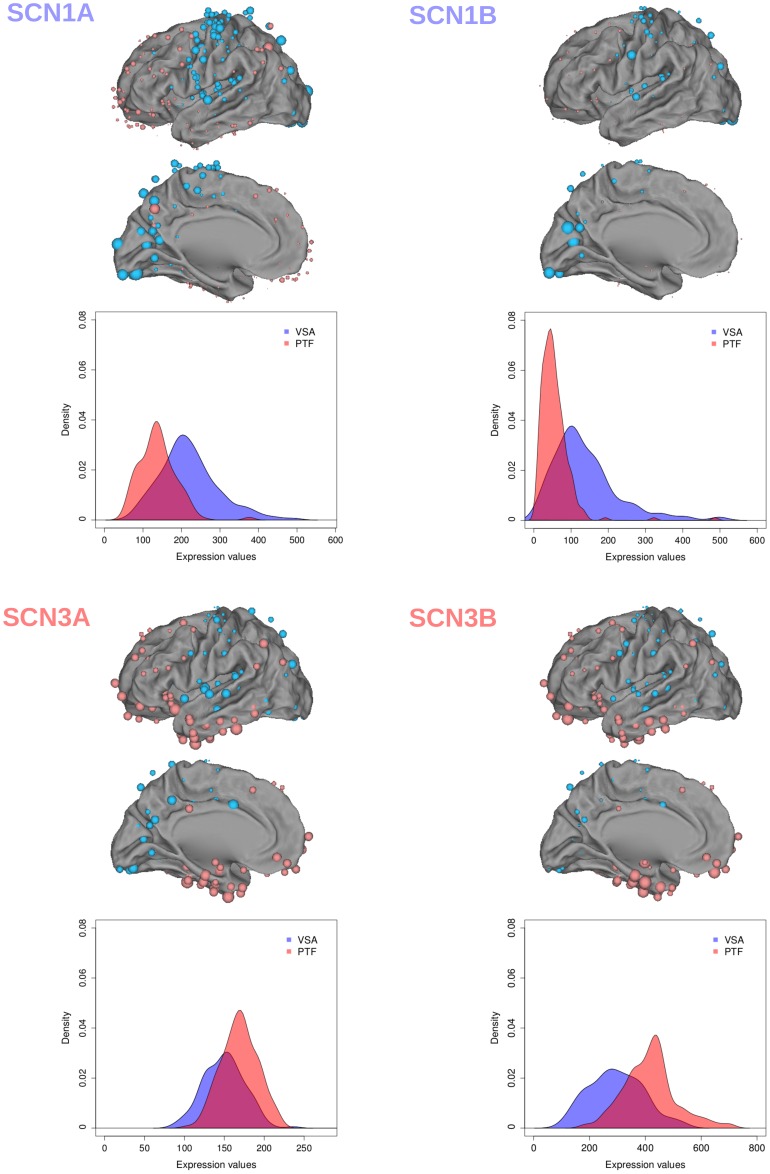
Example of cortical mapping of gene expression with extreme bootstrap ratios in PTF or VSA. Top. Cortical mapping of the expression of the 4 SCN genes with the highest bootstrap ratios in PTF or VSA: each dot represents one of the 394 cortical regions used in the analysis. The color depends upon the a priori assignment of each region the VSA-ring (in blue) to the PTF-ring (in red). The size of a dot is proportional to the expression of the gene under consideration for the cortical region represented by this dot. Bottom. For each gene, we plot the histogram of the number of regions as a function of gene expression intensities for the VSA (blue) and PTF (red) rings.

The most characteristic temporal properties associated to the extreme genes (i.e., preferentially expressed in one of the two rings) coding for ionic channels and transmitter release proteins are detailed in the discussion. The families belonging to the ionic channels neuronal function class present a similar characteristic: their members are spread from extreme VSA to extreme PTF and comprise two groups of genes, which discriminate strongly between the VSA and PTF regions. In particular, extreme members of the sodium channel (SCN) family have some of the highest bootstrap ratios. For the sodium channels family on the VSA side, we found SCN1B (*BT* = 12.10) and SCN1A (*BT* = 11.17) while on the other extreme preferentially expressed in the PTF ring (and not present in the 938 genes) we found SCN3B (*BT* = –13.55) and SCN3A (*BT* = –10.62). To illustrate the relation between a high bootstrap ratio and the cortical distribution for a gene (preferential distribution on a ring) we show two examples in Fig. 8.

Fig. 8 shows the extreme members belonging to the SCN family (i.e., SCN1A and SCN1B vs. SCN3A and SCN3B). For the potassium channel family we found, with the highest bootstrap ratio in VSA, KCNA1 (*BT* = 11.49) and on the other side we found KCNG1 (*BT* = –16.80). For the calcium channel family, CACNA2D2 had the highest bootstrap ratio in the VSA ring (*BT* = 7.12) while CACNG3 had the highest bootstrap ratio in the PTF ring (*BT* = –9.20). For the synaptotagmin family we found, on the VSA side, SYT2 with an extremely high boot ratio (*BT* = 13.49). By contrast, on the PTF side we found SYT5 (*BT* = –8.52). For the complexin family, on the VSA extreme, we found CPLX1 (*BT* = 9.29). Conversely, on the PTF side we found as the most extreme gene CPLX3 (*BT* = –9.37). Finally, for the VAMP family we found VAMP1 preferentially expressed in the VSA ring with a *BT* = 13.53 while VAMP2 was preferentially expressed in the PTF ring with a *BT* = –5.38.

## Discussion

In this paper we correlated the topographic organization of gene expressions with the anatomo-functional organization of the cerebral cortex. To evaluate gene expression we used the Allen Human Brain Atlas [38] of the whole transcriptome because these data constitute the unique set that covers the entire cortex for 21,000 genes. We first focused our analysis on the subset of 938 genes that were selected in [7] because they had the largest variance of expression over the cortex. This first analysis revealed some families of genes important for basic neuronal information processing. We then decided to specifically analyze two classes of genes: 1) genes coding for ionic channels and 2) genes coding for proteins involved in transmitter release. For the cognitive networks, we used a model–called the dual intertwined rings architecture [3]–that integrates in a common framework large cognitive [2], resting state [16], [17], anatomical networks, and functional connectivity. Most published results dealing with the organization of RSNs present patterns that are compatible with this model [1], [22], [23], [51], [52]. According to the two-ring model, the cortex is organized in two main regions called the VSA and PTF rings that mainly differ in the temporal integration of the information that they process: The VSA ring comprises brain regions involved in immediate real-time information processing of sensory, motor, and bimodal information, whereas the PTF ring integrates systems dedicated to multi-temporal processes (e.g., language, episodic memory, social interactions, self) with systems dedicated to emotions, basic biological needs, and rhythms. Evidence for this regional temporal specialization has also been found in experiments using single cell recording in non-human primates [35] and in empirically based mathematical models of the human cortex [36].

Our first result indicates that the cortical organization in two rings constitutes the major source of genetic variation in the cortex. This result shows that cortical gene expression spontaneously organizes the cortex into two rings. This result extends a previous result [7] that showed–using a principal component analysis–that the opposition of primary vs associative regions was important for the organization of genetic expression, but in addition, the present results suggest that the cortex is organized as a two-rings topography comprising two sets of functional regions differentiated by their genetic expression. A second result showed that the profiles of gene expression of a cortical region could reliably be used to assign this region to one ring. However, certain cortical regions were not correctly classified and, interestingly most of the ill-assigned regions were localized on the border between the two rings. The critical importance of the ring model is confirmed by the third result that shows that gene expression is strongly correlated within the two rings and anti-correlated between them.

**Figure 9:**
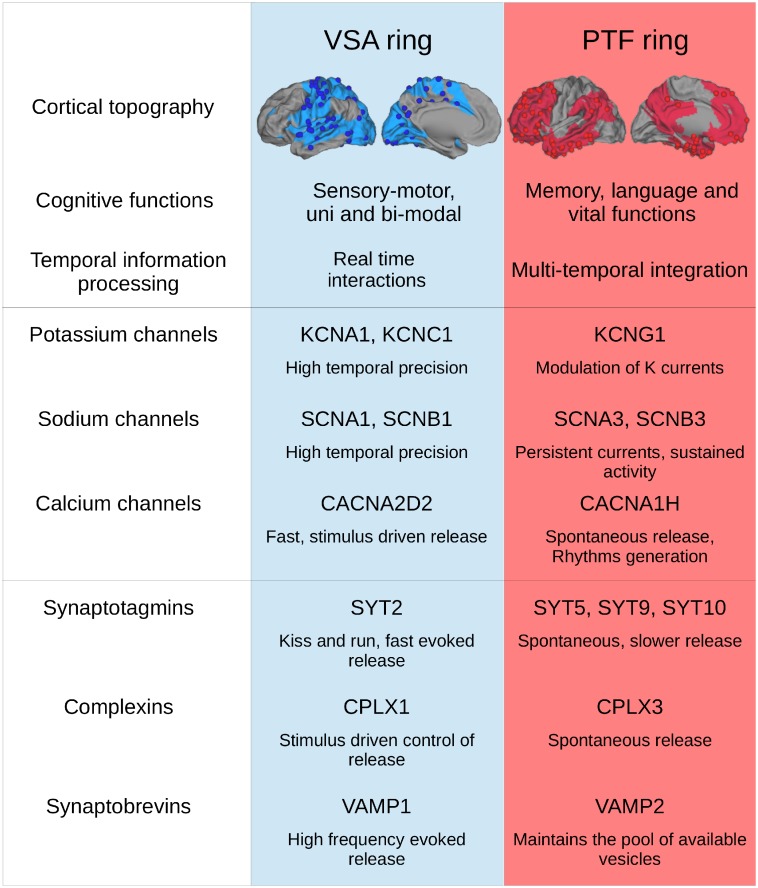
Summary of the temporal properties of proteins that most differentiate between the two rings and their correspondence with the preferred information processing modes of the rings.

We found in our first analysis using 938 genes that many proteins coded by genes that most differentiated between cortical regions belong to two classes of proteins involved in neuronal information processing namely ionic channels and neurotransmitter release. We then decided to specifically focus on the systematic study of these two families of genes comprising six families of genes (see Table 1). In the following section, we identify the genes with the highest bootstrap ratios (from the DiCA analysis) and discuss the cellular properties controlled by these genes in relation with the type of information processing performed by the two rings (see Fig. 9 for a summary of this section).

### Sodium and potassium channels

The sodium (SCN) and the potassium (KCN) channel families are distributed in the two rings. In fact, SCN and KCN family members turn out to be spread ranging from extremely PTF to extremely VSA expression with high bootstrap ratios on both sides. In the SCN family, we found the highest bootstrap ratios on the VSA side for SCN1A (*BT* = 11.17) and SCN1B (*BT* = 12.10). The sodium channel, voltage-gated Nav1.1, is essential for the generation and propagation of action potentials, with a large alpha subunit encoded by SCN1A, and smaller beta subunits, encoded by SCN1B, important for its fast inactivation kinetics [53]. These two subunits play a critical role in the temporal precision of neuronal information processing. For the highest expression of K channels in VSA, we found KCNA1 (*BT* = 11.49). This gene codes for a subunit responsible for currents Kv1.1, which, in turn, play a major role in maintaining action potential temporal precision: This gene is expressed in neurons that fire temporally precise action potentials [54], in particular for processing rapid acoustic stimuli, animal vocalizations, human speech [55], sound localization (which depends on timing occurring within a submillisecond epoch) [56]. This potassium channel subunit is important for regulating a tight input-output correspondence and temporal synchrony [57]. We thus found, more expressed in the VSA ring, genes (SCNA1, SCNB1, KCNA1) which control the temporal precision of neuronal activation, and these ionic channel properties match the data-driven, real-time information processing in the VSA ring underlying sensorimotor interactions. Conversely, on the PTF side, we found for sodium channels an overexpression of both SCN3A (*BT* = –10.62) and SCN3B (*BT* = –13.55) (alpha and beta subunits of Nav1.3). The beta3-subunit influences the temporal properties of sodium channels in such a way that they stay open and active for a longer time [58], and favor persistent sodium currents and sustained activation of the neuron. [59]–[62]. The next gene with the highest bootstrap ratio, SCN9A (*BT* = –10.66) codes for sodium channels Nav1.9 and contributes also to foster a persistent sodium current [63]. For the KCN family, more expressed on the PTF side, we found the genes KCNF1 (Kv5.1, *BT* = *–*6.25) and KCNG1 (Kv6.1, *BT* = *–*16.80) that code for proteins that are electrically silent Kv (KvS) potassium subunits which modulate the Kv2.1 channel, the major delayed rectifier channel expressed in most cortical pyramidal neurons, and slow its activation, inactivation and deactivation kinetics (reviewed in [64]–[65]). We found other genes overexpressed in the PTF ring–such as KCNMB4 (*BT* = –9.21)–which have similar effects. We found also KCNN3 (*BT* = *–*6.56) which induces a sustained activation of dopamine neurons [66], KCNJ1 (*BT* = –7.51) [67] which generates rhythmic activation and spontaneous activity, and KCNA4 (*BT* = –7.17) which regulates long term circadian rhythms [68]. We thus found, more expressed in the PTF ring, genes which facilitate persistent currents (SCN3A and SCN3B), prolonged effects of input activities, (KCNF1, KCNG1, KCNMB4, KCNN3), and spontaneous and rhythmic activation (KCNJ1, KCNA4): All these temporal neuronal properties match the multi-temporal processing in the PTF ring, contrasting with the high precision timing control of SCN1A, SCN1B and KCNA1 in the VSA ring. However, the precise role of these genes in the different regions of the PTF ring remains to be elucidated.

### Calcium channels

We found also calcium channels with a preferential distribution either in the VSA or the PTF ring. Calcium [Ca(2+)] channels initiate release of neurotransmitters at synapses, from the timescale of milliseconds to minutes, in response to the frequency of action potentials [69]. Mostly expressed in the VSA ring we found CACNA2D2 (*BT* = 7.16). This subunit causes faster activation and inactivation kinetics [70] of calcium channels and drive exocytosis with an increased release probability, making synapses more efficient at driving neurotransmitter release [71]. Mutations of CACNA2D2 result in slow inactivation of calcium channels and a prolonged calcium entry during depolarization [72]. The control of input-output timing and quantification of evoked transmitter release is requested for data-driven real-time processing in the VSA ring underlying sensorimotor functions. Conversely, on the PTF side, we found CACNA1H (*BT* = *–*7.71). This gene encodes a syntaxin-1A/Cav3.2 T-type calcium channel which controls low-threshold exocytosis in an action potential-independent manner [73]–[74], and can trigger the release of neurotransmitters at rest. This produces low-threshold burst of action potentials for the genesis of neuronal rhythms. Similarly, CACNG3 strongly expressed on PTF (*BT* = –9.20) shows multiple long-lived components [75] which can facilitate prolonged activation of neurons. The spontaneous, prolonged, or rhythmic release, matches the multi-temporal processing in the PTF ring for vital needs, memory, and language, compared to the high fidelity evoked processing in the VSA ring.

### Synaptotagmins

Central synapses operate neurotransmission in several modes: synchronous/fast neurotransmission–neurotransmitters release is tightly coupled to action potentials–and spontaneous neurotransmission where small amounts of neurotransmitter are released without being evoked by an action potential [76]. Synaptotagmins (SYTs) are abundant membrane proteins [77], [78], with at least 16 isoforms in mammals, which influence the kinetics of exocytotic fusion pores and the choice of release mode between full-fusion and “kiss-and-run” (partial fusion) of vesicles with the presynaptic membrane [77], [79], [80]. Kiss-and-run allows neurons to respond to high-frequency inputs mediating tight millisecond coupling of an action potential to neurotransmitter release [78]. The synaptotagmin most strongly associated to VSA is SYT2 (*BT* = 13.49) which controls the kiss-and-run behavior of vesicles and temporal accuracy of transmitter release [80], [81] for example in the auditory system or the neuromuscular junction. The kiss-and-run mode of SYT2 matches the real-time data-driven processing mode of VSA.

By contrast, we found more expressed in the PTF ring, the synaptotagmin V. SYT5 (BT = –8.52) which promotes the fusion of vesicles with a slower binding and is targeted to dense-core vesicles [821] that undergo Calcium-dependent exoocytosis of various neurohormones and neuropeptides. The SYT5 preferred expression in the PTF ring is compatible with the multi-temporal processing mode of the PTF ring and contrasts with other synaptotagmins (e.g., SYT2) which favor high temporal precision in response to evoked stimuli (SYT2) and are therefore more expressed in VSA.

### Complexins

We also studied the complexin (CPLX) family whose members assist synaptotagmin by activating and/or clamping the core fusion machinery of exocytosis of vesicles controlling either spontaneous or evoked neurotransmitter release [83]–[85]. In the complexin family, we found that CPLX1 (*BT* = 9.29) was strongly expressed in the VSA ring. CPLX1 is important for the tight time coupling (of the order of a millisecond) of the action potential to neurotransmitter release [86] while suppressing spontaneous synaptic vesicle exocytosis driven by low levels of endogenous neural activity [87]. Here again, a fast evoked release mechanism matches the real-time processing mode of the VSA ring. Conversely, on the PTF side, we found as the most extreme gene CPLX3 (*BT* = –9.37), which is an activator of spontaneous exocytosis [86]. Furthermore, the molecular structural differences between Complexins 1 and 3 have been studied in relation with their two different functional roles, where the C-terminal sequence of CPLX1 facilitates evoked release whereas the C-terminal sequence of CPLX3 facilitates spontaneous release. The preferential expression of CPLX1 and CPLX3, which induces either evoked or spontaneous release, matches both the real-time data-driven processing in VSA and the goal-driven multi-temporal integration in PTF that is based on more spontaneous activation.

### Synaptobrevins

Our results also show a differential expression of synaptobrevins (VAMP vesicle-associated membrane protein) in VSA or PTF. This protein family plays an important role in vesicle docking and maintenance controlling the rate of release. VAMP1 (*BT = *13.53) gene is strongly expressed in the VSA ring–a pattern consistent with previous results showing that these proteins are important for the fast processing of sensory stimuli such as auditory stimuli [88]. Results have shown that VAMP1 (Synaptobrevin 1) is more important for evoked release [89], while Synaptobrevin 2 (VAMP2, *BT* = *–*5.38) seems to have a more general role in maintaining a pool of available vesicles. Here again this differentiation matches the importance of precise control of evoked release in the VSA ring.

In summary (see Fig. 9), our results showed co-expression in VSA of a set of genes which favor high temporal precision of fast evoked neural transmission: Sodium channels SCNA1, SCNB1 and potassium channels KCNA1, and proteins facilitating fast evoked transmitter release: calcium channels CACNA2D2, Synaptotagmin SYT2, Complexin CPLX1, Synaptobrevin VAMP1. Conversely the results showed in PTF co-expression of a set of genes responsible for slower, sustained, or rhythmic activation: Sodium channels SCNA3, SCNB3, SCN9A potassium channels KCNF1, KCNG1, KCNMB4, KCNJ1, KCNA4, KCNN3 together with genes that facilitate spontaneous transmitter release: calcium channel CACNA1H, Synaptotagmin SYT5, Complexin CPLX3, and Synaptobrevin VAMP2. The results showed that there is a strong congruence between the preferential expression of a subset of genes, the temporal properties of the proteins that they code, and the temporal processing modes of the two rings underlying their sensorimotor and cognitive functions.

## Conclusions

The expression of the 938 genes most differentially expressed across the cortex organized the cortex into two sets of regions (called rings) that match two–previously described–large scale human functional cortical networks. The first ring processes real time sensory-motor information whereas the second ring processes multi-scale temporal information such as language, memory, or vital rhythms. The systematic study of families of genes coding for ionic channels and transmitter release proteins showed strong congruence between the preferential expression of genes, the temporal properties of the proteins they code at the cell level, and the temporal processing modes of these two large scale networks.

## Supporting Information

S1 Fig **Differential distribution of gene expression: CA analysis on H0351.2002.**
(TIFF)

S2 Fig **DiCA analysis: regions factor scores histogram for H0351.2002.** We plot the histogram of the factor score values–obtained for the 337 regions by the DiCA analysis–as a function of the number of regions *a priori* assigned to the VSA (blue) or the PTF (red) ring.
(TIFF)

S3 Fig **Brain H0351.2002 (161 genes) DiCA bootstrap ratios.** In blue are represented genes with significant positive bootstrap ratios (*BT*>4.00) associated with the VSA ring and in red, genes with significant negative bootstrap ratios (*BT*<–4.00) associated with the PTF ring. For each family, extreme genes are identified. These genes are the most preferentially expressed in either VSA or PTF.
(TIFF)

S1 Table **Brain H0351.2001 CA regions factor scores for Dimensions 1 and 2 and bootstrap ratios for Dimension 1.**
(CSV)

S2 Table **Brain H0351.2002 CA regions factor scores for Dimensions 1 and 2 and bootstrap ratios for Dimension 1.**
(CSV)

S3 Table **Brain H0351.2001 (938 genes) DiCA confusion matrix, fixed effect model.** Confusion matrix for the fixed effect assignments of the ROIs to the rings. The columns represent the *a priori* assignment and the rows the actual (*a posteriori*) model assignment. Diagonal entries represent correct assignments.
(DOC)

S4 Table **Brain H0351.2002 (983 genes) DiCA confusion matrix, fixed effect model.** Confusion matrix for the fixed effect assignments of the ROIS to the rings. The columns represent the *a priori* assignment and the rows the actual (*a posteriori*) model assignment. Diagonal entries represent correct assignments.
(DOC)

S5 Table **Brain H0351.2001 (938 genes) DiCA confusion matrix, random effect model.** Confusion matrix for the random effect assignment of the ROIS to the rings. The columns represent the *a priori* assignment and the rows the (*a posteriori*) model assignment. Diagonal entries represent correct assignments.
(DOC)

S6 Table **Brain H0351.2002 (983 genes) DiCA confusion matrix, random effect model.** Confusion matrix for the random effect assignment of the ROIS to the rings. The columns represent the *a priori* assignment and the rows the (*a posteriori*) model assignment. Diagonal entries represent correct assignments.
(DOC)

S7 Table **Brain H0351.2001 DiCA: Bootstrap ratios for the 938 genes.**
(CSV)

S8 Table **Brain H0351.2002 DiCA: Bootstrap ratios for the 983 genes.**
(CSV)

S9 Table **Brain H0351.2001 (161 genes) DiCA confusion matrix, fixed effect model.** Confusion matrix for the fixed effect assignments of the ROIS to the rings. The columns represent the *a priori* assignment and the rows the actual (*a posteriori*) model assignment. Diagonal entries represent correct assignments.
(DOC)

S10 Table **Brain H0351.2001 (161 genes) DiCA confusion matrix, random effect model.** Confusion matrix for the random effect assignment of the ROIS to the rings. The columns represent the *a priori* assignment and the rows the (*a posteriori*) model assignment. Diagonal entries represent correct assignments.
(DOC)

S11 Table **Brain H0351.2002 (161 genes) DiCA confusion matrix, fixed effect model.** Confusion matrix for the fixed effect assignments of the ROIS to the rings. The columns represent the *a priori* assignment and the rows the actual (*a posteriori*) model assignment. Diagonal entries represent correct assignments.
(DOC)

S12 Table **Brain H0351.2002 (161 genes) DiCA confusion matrix, random effect model.** Confusion matrix for the random effect assignment of the ROIS to the rings. The columns represent the *a priori* assignment and the rows the (*a posteriori*) model assignment. Diagonal entries represent correct assignments.
(DOC)

S13 Table **Brains H0351.2001 and H0351.2002 DiCA: Bootstrap ratios for the 161 genes.**
(CSV)

## References

[1] Fox MD, Corbetta M, Snyder AZ, Vincent JL, Raichle ME (2006) Spontaneous neuronal activity distinguishes human dorsal and ventral attention systems. Proc Natl Acad Sci USA 103:10046–10051. 16788060 10.1073/pnas.0604187103 PMC1480402

[2] Laird AR, Fox PM, Eickhoff SB, Turner JA, Ray KL, et al (2011) Behavioral interpretations of intrinsic connectivity networks. J Cogn Neurosci 23:4022–4037. 21671731 10.1162/jocn_a_00077 PMC3690655

[3] Mesmoudi S, Perlbarg V, Rudrauf D, Messe A, Pinsard B, et al (2013) Resting state networks’ corticotopy: the dual intertwined rings architecture. PLoS ONE 8:e67444. 23894288 10.1371/journal.pone.0067444 PMC3722222

[4] Sansom SN, Livesey FJ (2009) Gradients in the brain: the control of the development of form and function in the cerebral cortex. Cold Spring Harb Perspect Biol 1:a002519. 20066088 10.1101/cshperspect.a002519 PMC2742095

[5] Schlaggar BL (2011) Mapping genetic influences on cortical regionalization. Neuron 72:499–501. 22099452 10.1016/j.neuron.2011.10.024 PMC3226785

[6] Goyal MS, Hawrylycz M, Miller JA, Snyder AZ, Raichle ME (2014) Aerobic glycolysis in the human brain is associated with development and neotenous gene expression. Cell Metab 19:49–57. 24411938 10.1016/j.cmet.2013.11.020 PMC4389678

[7] Hawrylycz MJ, Lein ES, Guillozet-Bongaarts AL, Shen EH, Ng L, et al (2012) Ananatomically comprehensive atlas of the adult human brain transcriptome. Nature 489:391–399. 22996553 10.1038/nature11405 PMC4243026

[8] Zeng H, Shen EH, Hohmann JG, Oh SW, Bernard A, et al (2012) Large-scale cellular-resolution gene profiling in human neocortex reveals species-specific molecular signatures. Cell 149:483–496. 22500809 10.1016/j.cell.2012.02.052 PMC3328777

[9] Grange P, Bohland JW, Okaty BW, Sugino K, Bokil H, et al (2014) Cell-type-based model explaining coexpression patterns of genes in the brain. Proc Natl Acad Sci USA 111:5397–5402. 24706869 10.1073/pnas.1312098111 PMC3986204

[10] Bohland JW, Bokil H, Pathak SD, Lee CK, Ng L, et al (2010) Clustering of spatial gene expression patterns in the mouse brain and comparison with classical neuroanatomy. Methods 50:105–112. 19733241 10.1016/j.ymeth.2009.09.001

[11] French L, Pavlidis P (2011) Relationships between gene expression and brain wiring in the adult rodent brain. PLoS Comput Biol 7:e1001049. 21253556 10.1371/journal.pcbi.1001049 PMC3017102

[12] French L, Tan, Pavlidis P (2011) Large-Scale Analysis of Gene Expression and Connectivity in the Rodent Brain: Insights through Data Integration. Front Neuroinform 5:12. 21863139 10.3389/fninf.2011.00012 PMC3149147

[13] Grange P, Mitra (2011) Statistical analysis of co-expression properties of sets of genes in the mouse brain. ArXiv. Available: http://arxiv.org/abs/1111.6200. Accessed 12 September 2014.

[14] Wolf L, Goldberg C, Manor N, Sharan R, Ruppin E (2011) Gene expression in the rodent brain is associated with its regional connectivity. PLoS Comput Biol 7:e1002040. 21573208 10.1371/journal.pcbi.1002040 PMC3088660

[15] Goel P, Kuceyeski A, LoCastro E, Raj A (2014) Spatial patterns of genome-wide expression profiles reflect anatomic and fiber connectivity architecture of healthy human brain. Hum Brain Mapp 35:4204–4218. 24677576 10.1002/hbm.22471 PMC4283562

[16] Yan H, Zuo XN, Wang D, Wang J, Zhu C, et al (2009) Hemispheric asymmetry in cognitive division of anterior cingulate cortex: a resting-state functional connectivity study. Neuroimage 47:1579–1589. 19501172 10.1016/j.neuroimage.2009.05.080

[17] Smith SM, Fox PT, Miller KL, Glahn DC, Fox PM, et al (2009) Correspondence of the brain’s functional architecture during activation and rest. Proc Natl Acad Sci USA 106:13040–13045. 19620724 10.1073/pnas.0905267106 PMC2722273

[18] Cauda F, Geminiani GC, Vercelli A (2014) Evolutionary appearance of von Economo’s. neurons in the mammalian cerebral cortex. Front Hum Neurosci 8:104. 24672457 10.3389/fnhum.2014.00104 PMC3953677

[19] Cauda F, Palermo S, Costa T, Torta R, Duca S, et al (2014) Gray matter alterations in chronic pain: A network-oriented meta-analytic. approach. Neuroimage Clin 4:676–686. 24936419 10.1016/j.nicl.2014.04.007 PMC4053643

[20] Thirion B, Varoquaux G, Grisel O, Poupon C, Pinel P (2014) Principal Component Regression predicts functional responses across individuals. Med Image Comput Comput Assist Interv 8674:741–748. 10.1007/978-3-319-10470-6_92 25485446

[21] Premi E, Cauda F, Gasparotti R, Diano M, Archetti S, et al (2014) Multimodal FMRI resting-state functional connectivity in granulin mutations: the case of fronto-parietal dementia. PLoS One 9:e106500. 25188321 10.1371/journal.pone.0106500 PMC4154688

[22] Doucet G, Naveau M, Petit L, Delcroix N, Zago L, et al (2011) Brain activity at rest: a multiscale hierarchical functional organization. J Neurophysiol 105:2753–2763. 21430278 10.1152/jn.00895.2010

[23] Yeo BT, Krienen FM, Sepulcre J, Sabuncu MR, Lashkari D, et al (2011) The organization of the human cerebral cortex estimated by intrinsic functional connectivity. J Neurophysiol 106:1125–1165. 21653723 10.1152/jn.00338.2011 PMC3174820

[24] Sepulcre J, Sabuncu MR, Yeo TB, Liu H, Johnson KA (2012) Stepwise connectivity of the modal cortex reveals the multimodal organization of the human brain. J Neurosci 32(31):10649–1066. 22855814 10.1523/JNEUROSCI.0759-12.2012 PMC3483645

[25] Milner B, Petrides M, Smith M (1985) Frontal lobes and the temporal organization of memory. Hum Neurobiol 4:137–142. 4066424

[26] Fuster JM (1989) The Prefrontal Cortex: Anatomy, Physiology, and Neuropsychology of the Frontal Lobe. New York: Raven Press.

[27] Gusnard DA, Raichle ME (2001) Searching for a baseline: functional imaging and the resting human brain. Nat Rev, Neurosci 2:685–694. 11584306 10.1038/35094500

[28] Raichle ME, MacLeod AM, Snyder AZ, Powers WJ, Gusnard DA, et al (2001) A default mode of brain function. Proc Natl Acad Sci USA 98:676–682. 11209064 10.1073/pnas.98.2.676 PMC14647

[29] Saxe R, Kanwisher N (2003) People thinking about thinking people. The role of the temporo-parietal junction in “theory of mind”. Neuroimage 19:1835–1842. 12948738 10.1016/s1053-8119(03)00230-1

[30] Schacter DL, Addis DR, Buckner RL (2007) Remembering the past to imagine the future: the prospective brain. Nat Rev Neurosci 8:657–661. 17700624 10.1038/nrn2213

[31] Buckner RL, Andrews-Hanna JR, Schacter DL (2008) The brain’s default network: anatomy, function, and relevance to disease. Ann N Y Acad Sci 1124:1–38. 18400922 10.1196/annals.1440.011

[32] Binder JR, Desai R, Conant LL, Graves WW (2009) Where is the semantic system? A critical review and meta-analysis of 120 functional neuroimaging studies. Cereb Cortex 19:2767–2796. 19329570 10.1093/cercor/bhp055 PMC2774390

[33] Fuster JM (2006) The cognit: a network model of cortical representation. Int J Psychophysiol 60:125–132. 16626831 10.1016/j.ijpsycho.2005.12.015

[34] Fuster JM, Bressler SL (2012) Cognit activation: a mechanism enabling temporal integration in working memory. Trends Cogn Sci 16:207–218. 22440831 10.1016/j.tics.2012.03.005 PMC3457701

[35] Murray JD, Bernacchia A, Freedman DJ, Romo R, Wallis JD, et al (2014) A hierarchy of intrinsic timescales across primate cortex. Nature Neurosci 17:1161–1163. 10.1038/nn.3862 PMC4241138 25383900

[36] Kiebel SJ, Daunizeau J, Friston KJ (2008) A hierarchy of time-scales and the brain. PLoS Comput Biol 4:e1000209. 19008936 10.1371/journal.pcbi.1000209 PMC2568860

[37] Luria AR (1980) Higher Cortical Functions in Man. New York: Basic Books.

[38] Jones AR, Overly CC, Sunkin SM (2009) The Allen Brain Atlas: 5 years and beyond. Nat Rev Neurosci 10:821–828. 19826436 10.1038/nrn2722

[39] Lebart L, Morineau A, Warwick K (1984) Multivariate Descriptive Statistical Analysis. London: Wiley. 231 p.

[40] Greenacre M (1984) Theory and applications of correspondence analysis. London: Academic. Press. 364 p.

[41] Weller SC, Romney AK (1990) Metric scaling: Correspondence analysis. Thousand Oaks (CA): Sage. 96 p.

[42] Abdi H, Williams LJ (2010) Correspondence analysis. In NJ Salkind, DM, Dougherty, and B Frey, editors. Encyclopedia of Research Design. Thousand Oaks (CA): Sage. 267–278.

[43] Abdi H, Béra M (2014) Correspondence analysis. In R Alhajj and J Rokne editors, Encyclopedia of Social Networks and Mining. New York: Springer Verlag. 275–284.

[44] Abdi H (2007) Discriminant correspondence analysis. In NJ Salkind, DM, Dougherty, and B Frey, editors. Encyclopedia of Research Design. Thousand Oaks (CA): Sage. 270–275.

[45] Evan AC, Collins DL, Mills SR, Brown DE, Kelly RL, et al**.** (1993) 3D statistical neuroanatomical models from 305 MRI volumes. IEEE Nucl Sci Symp Conf Rec 1813–1817.

[46] Shankle WR, Romney AK, Landing BH, Hara J (1998) Developmental patterns in the cytoarchitecture of the human cerebral cortex from birth to 6 years examined by correspondence analysis. Proc Natl Acad Sci USA 95:4023–4028. 9520486 10.1073/pnas.95.7.4023 PMC19956

[47] Beaton D, Chin Fatt CR, Abdi H (2014) An ExPosition of multivariate analysis with the Singular Value Decomposition in R. Comput Stat Data Anal. 72:176–189.

[48] Abdi H, Dunlop JP, Williams LJ (2009) How to compute reliability estimates and display confidence and tolerance intervals for pattern classifiers using the Bootstrap and 3-way multidimensional scaling (DISTATIS). Neuroimage 45:89–95. 19084072 10.1016/j.neuroimage.2008.11.008

[49] Krishnan A, Williams LJ, McIntosh AR, Abdi H (2011) Partial Least Squares (PLS) methods for neuroimaging: A tutorial and review. Neuroimage 56:455–475. 20656037 10.1016/j.neuroimage.2010.07.034

[50] Abdi H (2007) Bonferroni and Sidak corrections for multiple comparisons. In NJ Salkind, editor, Encyclopedia of Measurement and Statistics. Thousand Oaks (CA): Sage. 103–107.

[51] Vincent JL, Kahn I, Snyder AZ, Raichle ME, Buckner RL (2008) Evidence for a frontoparietal control system revealed by intrinsic functional connectivity. J Neurophysiol 100:3328–3342. 18799601 10.1152/jn.90355.2008 PMC2604839

[52] Sepulcre J, Liu H, Talukdar T, Martincorena I, Yeo BT, et al (2010) The organization of local and distant functional connectivity in the human brain. PLoS Comput Biol 6:e1000808. 20548945 10.1371/journal.pcbi.1000808 PMC2883589

[53] Catterall WA (2014) Structure and function of voltage-gated sodium channels at atomic resolution. Exp Physiol 99:35–51. 24097157 10.1113/expphysiol.2013.071969 PMC3885250

[54] Gittelman JX, Tempel BL (2006) Kv1.1-containing channels are critical for temporal precision during spike initiation. J Neurophysiol 96:1203–1214. 16672305 10.1152/jn.00092.2005

[55] Ison JR, Allen PD (2012) Deficits in responding to brief noise offsets in Kcna1 −/− mice reveal a contribution of this gene to precise temporal processing seen previously only for stimulus onsets. J Assoc Res Otolaryngol 13:351–358. 22302114 10.1007/s10162-011-0312-1 PMC3346894

[56] Karcz A, Hennig MH, Robbins CA, Tempel BL, Rubsamen R, et al (2011) Low-voltage activated Kv1.1 subunits are crucial for the processing of sound source location in the lateral superior olive in mice. J Physiol 589:1143–1157. 21224222 10.1113/jphysiol.2010.203331 PMC3060593

[57] Allen PD, Ison JR (2012) Kcna1 gene deletion lowers the behavioral sensitivity of mice to small changes in sound location and increases asynchronous brainstem auditory evoked potentials but does not affect hearing thresholds. J Neurosci 32:2538–2543. 22396426 10.1523/JNEUROSCI.1958-11.2012 PMC3297021

[58] Cusdin FS, Nietlispach D, Maman J, Dale TJ, Powell AJ, et al (2010) The sodium channel {beta}3-subunit induces multiphasic gating in NaV1.3 and affects fast inactivation via distinct intracellular regions. J Biol Chem 285:33404–33412. 20675377 10.1074/jbc.M110.114058 PMC2963402

[59] Qu Y, Curtis R, Lawson D, Gilbride K, Ge P, et al (2001) Differential modulation of sodium channel gating and persistent sodium currents by the beta1, beta2, and beta3 subunits. Mol Cell Neurosci. 18:570–80. 11922146 10.1006/mcne.2001.1039

[60] Aman TK, Grieco-Calub TM, Chen C, Rusconi R, Slat EA, et al (2009) Regulation of persistent Na current by interactions between beta subunits of voltage-gated Na channels. J Neurosci 29:2027–2042. 19228957 10.1523/JNEUROSCI.4531-08.2009 PMC2667244

[61] Estacion M, Gasser A, Dib-Hajj SD, Waxman SG (2010) A sodium channel mutation linked to epilepsy increases ramp and persistent current of Nav1.3 and induces hyperexcitability in hippocampal neurons. Exp Neurol 224:362–368. 20420834 10.1016/j.expneurol.2010.04.012

[62] Cummins TR, Aglieco F, Renganathan M, Herzog RI, Dib-Hajj SD, et al (2001) Nav1.3 sodium channels: rapid repriming and slow closed-state inactivation display quantitative differences after expression in a mammalian cell line and in spinal sensory neurons. J Neurosci 21:5952–5961. 11487618 10.1523/JNEUROSCI.21-16-05952.2001 PMC6763143

[63] Vanoye CG, Kunic JD, Ehring GR, George AL Jr (2013) Mechanisms of sodium channel NaV1.9 potentiation by G-protein signaling. J Gen Physiol 141:193–202. 23359282 10.1085/jgp.201210919 PMC3557314

[64] Kramer JW, Post MA, Brown AM, Kirsch GE (1998) Modulation of potassium channel gating by coexpression of Kv2.1 with regulatory Kv5.1 or Kv6.1 alpha-subunits. Am J Physiol 274:C1501–1510. 9696692 10.1152/ajpcell.1998.274.6.C1501

[65] Bocksteins E, Snyders DJ (2012) Electrically silent Kv subunits: their molecular and functional characteristics. Physiology (Bethesda) 27:73–84. 22505664 10.1152/physiol.00023.2011

[66] Soden ME, Jones GL, Sanford CA, Chung AS, Guler AD, et al (2013) Disruption of dopamine neuron activity pattern regulation through selective expression of a human KCNN3 mutation. Neuron 80:997–1009. 24206670 10.1016/j.neuron.2013.07.044 PMC3840077

[67] Baccei ML (2014) Pacemaker Neurons and the Development of Nociception. Neuroscientist 20:197–202. 24510073 10.1177/1073858414521499 PMC4927414

[68] Granados-Fuentes D, Norris AJ, Carrasquillo Y, Nerbonne JM, Herzog ED (2012) I(A) channels encoded by Kv1.4 and Kv4.2 regulate neuronal firing in the suprachiasmatic nucleus and circadian rhythms in locomotor activity. J Neurosci 32:10045–10052. 22815518 10.1523/JNEUROSCI.0174-12.2012 PMC3752070

[69] Catterall WA, Few AP (2008) Calcium channel regulation and presynaptic plasticity. Neuron 59:882–901. 18817729 10.1016/j.neuron.2008.09.005

[70] Lacinova L, Klugbauer N (2004) Modulation of gating currents of the Ca(v)3.1 calcium channel by alpha 2 delta 2 and gamma 5 subunits. Arch Biochem Biophys 425:207–213. 15111129 10.1016/j.abb.2004.03.010

[71] Hoppa MB, Lana B, Margas W, Dolphin AC, Ryan TA (2012) α2δ expression sets presynaptic calcium channel abundance and release probability. Nature 486:122–125. 22678293 10.1038/nature11033 PMC3376018

[72] Edvardson S, Oz S, Abulhijaa FA, Taher FB, Shaag A, et al (2013) Early infantile epileptic encephalopathy associated with a high voltage gated calcium channelopathy. J Med Genet 50:118–123. 23339110 10.1136/jmedgenet-2012-101223

[73] Weiss N, Hameed S, Fernández-Fernández JM, Fablet K, Karmazinova M, et al (2012) A Ca(v)3.2/syntaxin-1A signaling complex controls T-type channel activity and low-threshold exocytosis. J Biol Chem 287:2810–2818. 22130660 10.1074/jbc.M111.290882 PMC3268438

[74] Cheong E, Shin HS (2013) T-type Ca2+ channels in normal and abnormal brain functions. Physiol Rev 93:961–992. 23899559 10.1152/physrev.00010.2012

[75] Shelley C, Farrant M, Cull-Candy SG (2012) TARP-associated AMPA receptors display an increased maximum channel conductance and multiple kinetically distinct open states. J Physiol 590:5723–5738. 22988139 10.1113/jphysiol.2012.238006 PMC3528987

[76] Chung C, Raingo J (2013) Vesicle dynamics: how synaptic proteins regulate different modes of neurotransmission. J Neurochem 126:146–154. 23517499 10.1111/jnc.12245

[77] Moghadam PK, Jackson MB (2013) The functional significance of synaptotagmin diversity in neuroendocrine secretion. Front Endocrinol (Lausanne) 4:124. 24065953 10.3389/fendo.2013.00124 PMC3776153

[78] Südhof TC (2013) Neurotransmitter release: the last millisecond in the life of a synaptic vesicle. Neuron 80:675–690. 24183019 10.1016/j.neuron.2013.10.022 PMC3866025

[79] Harata NC, Aravanis AM, Tsien RW (2006) Kiss-and-run and full-collapse fusion as modes of exo-endocytosis in neurosecretion. J Neurochem 97:1546–1570. 16805768 10.1111/j.1471-4159.2006.03987.x

[80] Zhang Q, Li Y, Tsien RW (2009) The dynamic control of kiss-and-run and vesicular reuse probed with single nanoparticles. Science 323:1448–1453. 19213879 10.1126/science.1167373 PMC2696197

[81] Kochubey O, Schneggenburger R (2011) Synaptotagmin increases the dynamic range of synapses by driving Ca2+-evoked release and by clamping a near-linear remaining Ca2+ sensor. Neuron 69:736–748. 21338883 10.1016/j.neuron.2011.01.013

[82] Saegusa C, Fukuda M, Mikoshiba K (2002) Synaptotagmin V is targeted to dense-core vesicles that undergo calcium-dependent exocytosis in PC12 cells. J Biol Chem 277:24499–24505. 12006594 10.1074/jbc.M202767200

[83] Jorquera RA, Huntwork-Rodriguez S, Akbergenova Y, Cho RW, Littleton JT (2012) Complexin controls spontaneous and evoked neurotransmitter release by regulating the timing and properties of synaptotagmin activity. J Neurosci 32:18234–18245. 23238737 10.1523/JNEUROSCI.3212-12.2012 PMC3530744

[84] Kaeser-Woo YJ, Yang X, Südhof TC (2012) C-terminal complexin sequence is selectively required for clamping and priming but not for Ca2+ triggering of synaptic exocytosis. J Neurosci 32:2877–2885. 22357870 10.1523/JNEUROSCI.3360-11.2012 PMC3742123

[85] Cao P, Yang X, Südhof TC (2013) Complexin activates exocytosis of distinct secretory vesicles controlled by different synaptotagmins. J Neurosci 33:1714–1727. 23345244 10.1523/JNEUROSCI.4087-12.2013 PMC3711587

[86] Yang X, Cao P, Südhof TC (2013) Deconstructing complexin function in activating and clamping Ca2+-triggered exocytosis by comparing knockout and knockdown phenotypes. Proc Natl Acad Sci USA 110:20777–20782. 24297916 10.1073/pnas.1321367110 PMC3870694

[87] Martin JA, Hu Z, Fenz KM, Fernandez J, Dittman JS (2011) Complexin has opposite effects on two modes of synaptic vesicle fusion. Curr Biol 21:97–105. 21215634 10.1016/j.cub.2010.12.014 PMC3026084

[88] Friedland DR, Eernisse R, Popper P (2008) Identification of a novel VAMP1 splice variant in the cochlear nucleus. Hear Res 243:105–112. 18655825 10.1016/j.heares.2008.06.009 PMC2562438

[89] Zimmermann J, Trimbuch T, Rosenmund C (2014) Synaptobrevin 1 mediates vesicle priming and evoked release in a subpopulation of hippocampal neurons. J Neurophysiol doi:10.1152/jn.00340.2014. 10.1152/jn.00340.2014 24944211

