# Supplementary figures and images for: Differences in Human Cortical Gene Expression Match the Temporal Properties of Large-Scale Functional Networks
Source: PLoS One. 2014 Dec 29;9(12):e115913. doi: 10.1371/journal.pone.0115913 (PMC4278769; doi:10.1371/journal.pone.0115913)

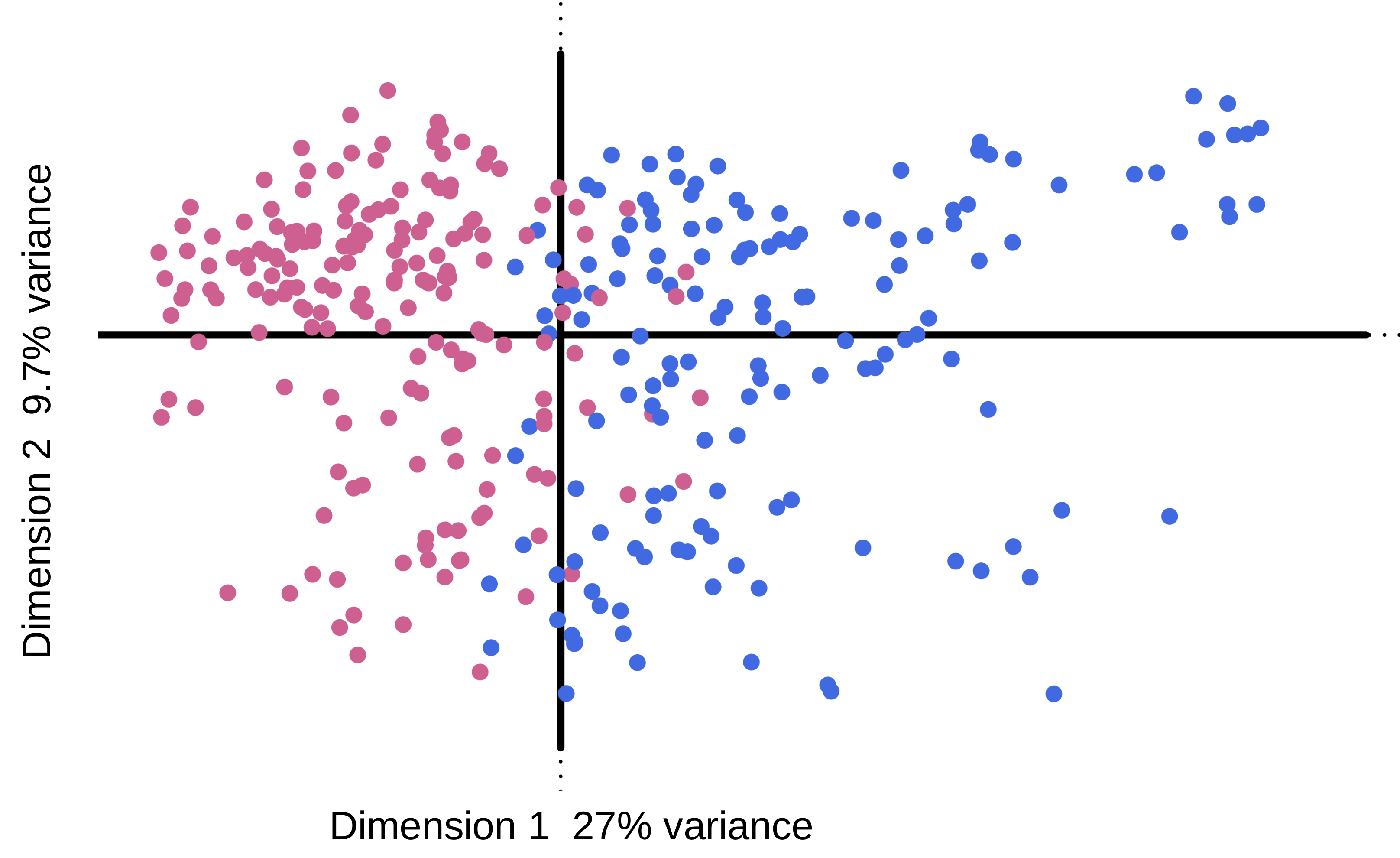

Supplement: S1 Fig — Differential distribution of gene expression: CA analysis on H0351.2002. (TIFF) [file pone.0115913.s001.tiff]

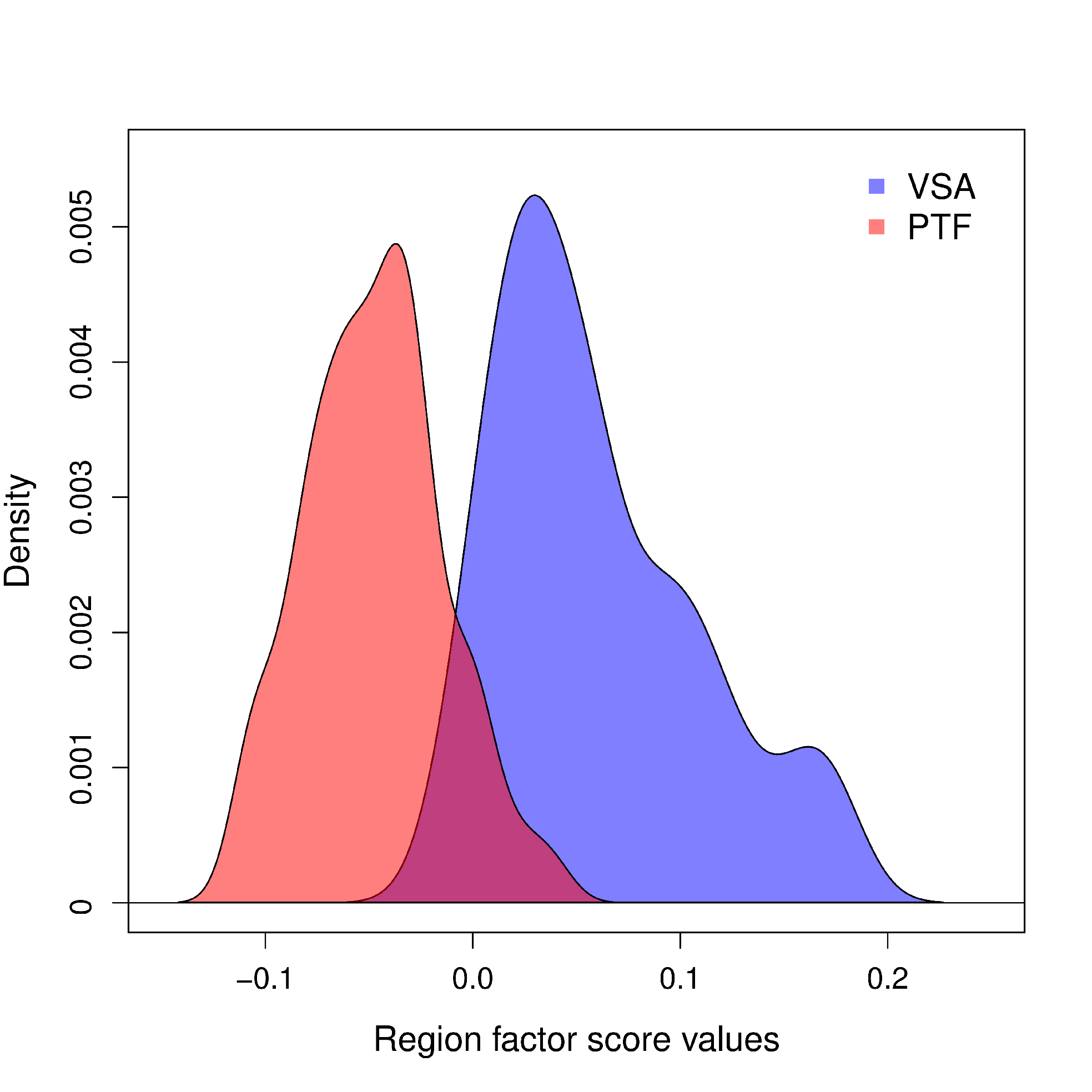

Supplement: S2 Fig — DiCA analysis: regions factor scores histogram for H0351.2002. We plot the histogram of the factor score values–obtained for the 337 regions by the DiCA analysis–as a function of the number of regions a priori assigned to the VSA (blue) or the PTF (red) ring. (TIFF) [file pone.0115913.s002.tiff]

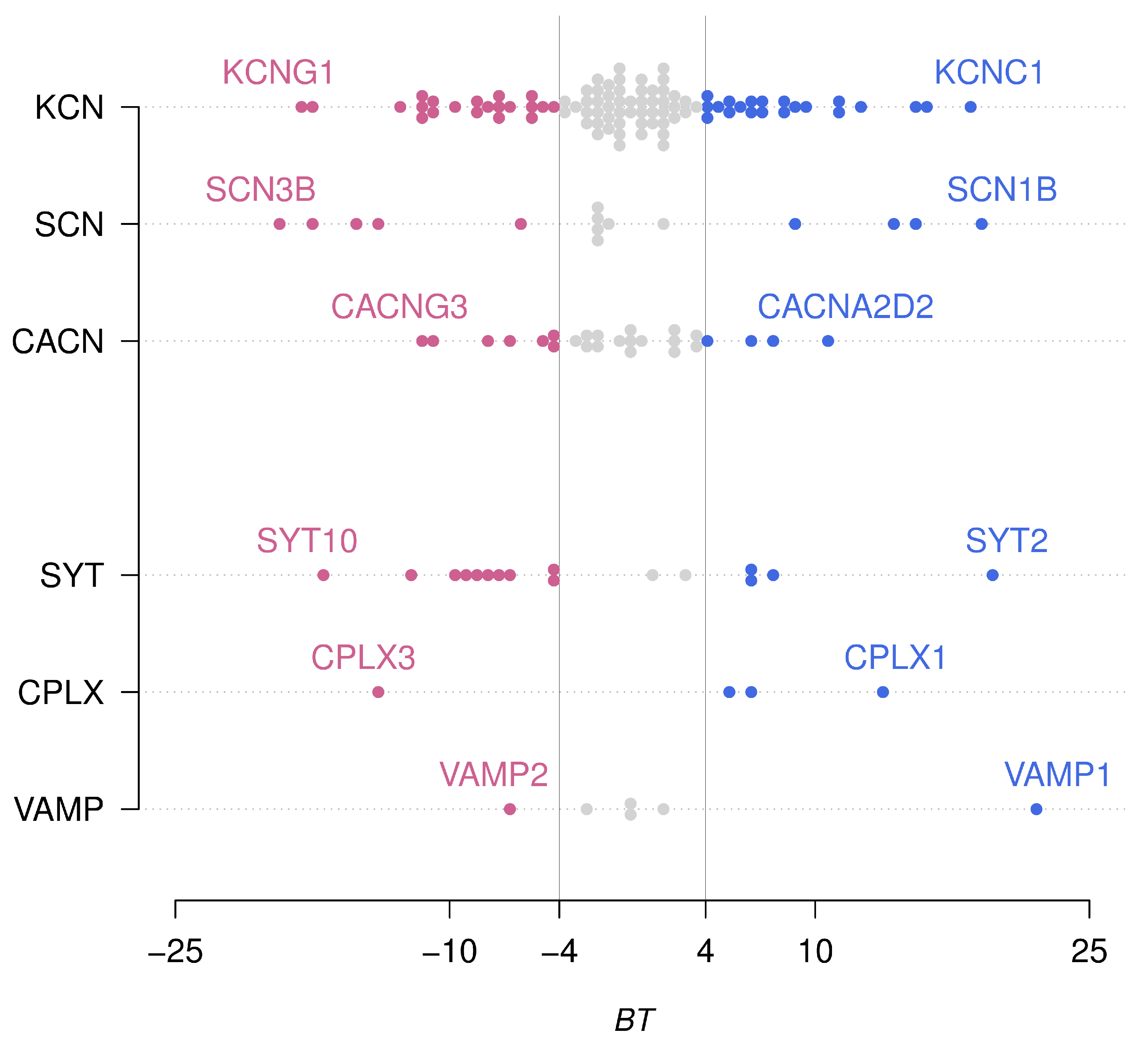

Supplement: S3 Fig — Brain H0351.2002 (161 genes) DiCA bootstrap ratios. In blue are represented genes with significant positive bootstrap ratios (BT>4.00) associated with the VSA ring and in red, genes with significant negative bootstrap ratios (BT<–4.00) associated with the PTF ring. For each family, extreme genes are identified. These genes are the most preferentially expressed in either VSA or PTF. (TIFF) [file pone.0115913.s003.tiff]
