# Supplementary material for: Differences in Human Cortical Gene Expression Match the Temporal Properties of Large-Scale Functional Networks
Source: PLoS One. 2014 Dec 29;9(12):e115913. doi: 10.1371/journal.pone.0115913 (PMC4278769; doi:10.1371/journal.pone.0115913)
Supplement: S4 Table — Brain H0351.2002 (983 genes) DiCA confusion matrix, fixed effect model. Confusion matrix for the fixed effect assignments of the ROIS to the rings. The columns represent the a priori assignment and the rows the actual (a posteriori) model assignment. Diagonal entries represent correct assignments. (DOC) [file pone.0115913.s007.doc]

**Table S4**

|  | VSA actual | PTF actual |
| --- | --- | --- |
| VSA predicted | 177 | 12 |
| PTF predicted | 10 | 138 |
