# Supplementary material for: Differences in Human Cortical Gene Expression Match the Temporal Properties of Large-Scale Functional Networks
Source: PLoS One. 2014 Dec 29;9(12):e115913. doi: 10.1371/journal.pone.0115913 (PMC4278769; doi:10.1371/journal.pone.0115913)
Supplement: S10 Table — Brain H0351.2001 (161 genes) DiCA confusion matrix, random effect model. Confusion matrix for the random effect assignment of the ROIS to the rings. The columns represent the a priori assignment and the rows the (a posteriori) model assignment. Diagonal entries represent correct assignments. (DOC) [file pone.0115913.s013.doc]

**Table S10**

|  | VSA actual | PTF actual |
| --- | --- | --- |
| VSA predicted | 130 | 35 |
| PTF predicted | 38 | 191 |
